# Comparison of the Efficacy of 1064‐ and 730‐nm Picosecond Lasers for Acquired Dermal Melanocytosis

**DOI:** 10.1111/jocd.70123

**Published:** 2025-03-15

**Authors:** Kento Takaya, Kazuo Kishi

**Affiliations:** ^1^ Department of Plastic and Reconstructive Surgery Keio University School of Medicine Tokyo Japan; ^2^ Etoile Regina Clinic Tokyo Japan

**Keywords:** acquired dermal melanocytosis, hyperpigmentation, laser therapy, melasma, picosecond laser

## Abstract

**Background:**

Acquired dermal melanocytosis (ADM) is a pigmentary lesion caused by melanocytes in the dermis. These conditions are refractory, and a consensus on treatment remains elusive.

**Aims:**

To compare the efficacy of 730‐ and 1064‐nm picosecond lasers in the treatment of ADM and to supplement the comparison with a literature review.

Patients:

We retrospectively examined patients who underwent picosecond laser therapy between April 2021 and February 2024. Treatments were performed three times with spot irradiation using a 730‐nm laser (3‐mm spot, 1.7–1.8 J/cm^2^; or 2‐mm spot, 2.5–3.25 J/cm^2^) and a 1064‐nm laser (3‐mm spot, 3.1–4.0 J/cm^2^). Two months after each procedure, patient satisfaction was evaluated using questionnaires, macroscopic findings were assessed using photographs, and melanin accumulation was analyzed using skin analysis software. Evaluations were conducted on a 4‐point scale (excellent, good, fair, and poor), with good or higher considered effective.

**Results:**

Seventy‐eight and 83 patients were assigned to the 730‐ and 1064‐nm groups, respectively. After three sessions, the 730‐nm group exhibited superior outcomes in terms of subjective symptoms (patient satisfaction), objective findings, and software analysis improvements. The incidence of hyperpigmentation was 15.4% in the 1064‐nm group and 14.5% in the 730‐nm group, with no significant differences observed in the rates of other complications.

**Conclusions:**

The use of a 730‐nm picosecond laser, which possesses high melanin selectivity, relatively deep penetration, and a short pulse width, suggests a potentially more effective treatment for ADM, compared to the effects of the conventional 1064‐nm wavelength, without increasing complications.

## 1 Introduction

Acquired dermal melanocytosis (ADM) is a pigmentary lesion characterized by bilateral grayish‐brown facial macules, which was first reported as an acquired bilateral nevus of Ota‐like macules (Hori's nevus) [1]. ADM typically presents bilaterally in individuals in their twenties and predominantly affects the zygomatic regions, although the lateral forehead, nasal, and auricular areas may also be involved [2]. ADM is more prevalent among women with darker skin tones, particularly those of East Asian descent, with 94.5% of the 549 reported cases originating from female patients [3]. Initially presenting as isolated brown macules, ADM evolves into confluent grayish‐brown patches or speckles. Its distribution pattern, poorly demarcated round grayish spots, and distinct colors differentiate it from melasma. The treatment of ADM poses challenges owing to its poor responsiveness to topical medications and cosmetic procedures such as superficial peeling. Lasers play a crucial role in managing these lesions due to their precise selectivity, depth of penetration, and ability to preserve the epidermis [4, 5, 6]. To date, the effectiveness of Q‐switched ruby lasers, Q‐switched alexandrite lasers, and scanning CO_2_ lasers has been reported in the treatment of ADM [7, 8, 9]. Notably, a Q‐switched Nd laser with a long 1064‐nm wavelength is widely employed for ADM treatment, as its longer wavelength allows for a deeper penetration with less absorption by epidermal melanin [10, 11, 12]. However, the pulse duration must be shorter than the thermal relaxation time of the target. Nanosecond laser pulses, while sufficient for tattoo removal, are often inadequate, leading to complications such as hyperpigmentation, hypopigmentation, tissue changes, and scarring. The recently developed picosecond lasers, capable of emitting even shorter picosecond energy pulses, address this issue by reducing unnecessary thermal diffusion to the surrounding structures and generating a greater photoacoustic effect [13]. In a split‐face randomized controlled trial conducted by Yu et al. involving 33 patients, the 755‐nm picosecond alexandrite laser demonstrated significant superiority over the nanosecond alexandrite laser in terms of pigment reduction, patient satisfaction, pain, healing time, and incidence of post‐inflammatory hyperpigmentation (PIH) 0.8. However, there is no consensus on the optimal wavelength. In this study, we treated ADM using both the conventional 1064‐nm wavelength and the 730‐nm wavelength, which has higher melanin selectivity and a shorter pulse width, aiming to compare their efficacy and patient satisfaction.

## 2 Materials and Methods

### 2.1 Patients

A retrospective analysis was conducted of patients who underwent PicoWay picosecond laser (Candela Corporation, Marlborough, MA) treatment for ADM at our two institutions between April 2021 and February 2024. We defined ADM as an acquired pigmented lesion that is blue‐gray to brownish‐red in color and appears in the area innervated by the first and second branches of the trigeminal nerve in the face (forehead to upper and lower eyelids, temples, nose to cheeks), based on experienced clinicians' judgment. We distinguished between lentigo solaris and ephilides by color. Melasma is a brown, relatively uniform, diffuse pigmented area, and if there is a uniform diffuse lesion around the ADM spot, it is considered that melasma is present. Even if the symptoms are the same as ADM, if there are pigmented spots on the sclera (whites of the eyes) or in the mouth, the condition is diagnosed as an ota nevus, and such patients are excluded.

The device features a pulse width of 339 ps for a 1064‐nm wavelength and 246 ps for the 730‐nm wavelength, a repetition rate of 1–10 Hz, and adjustable spot sizes and fluences. Treatments were administered three times using spot irradiation with the 730‐nm laser (3‐mm spot, 1.7–1.8 J/cm^2^; or 2‐mm spot, 2.5–3.25 J/cm^2^) and the 1064‐nm laser (3 mm spot, 3.1–4.0 J/cm^2^). Patients who had received pigment‐specific laser treatments for the target lesion within the past 3 months, those who had undergone concurrent laser or energy‐based treatments with the trial device, those who used topical skin‐lightening cream or ointment during the treatment period, or those without evaluable follow‐up digital photographs were excluded from the study.

### 2.2 Procedure

The treatment area was cleansed with a 1% chlorhexidine gluconate solution, and the procedure involved targeted irradiation of individual pigmented lesions using a trial device. Spot sizes were adjusted according to the lesion size, and the fluence was calibrated so that both wavelengths would induce a slight grayish‐white change (immediate whitening phenomenon) in the target lesion, ensuring that the surrounding normal skin remained unaffected. For the 1064‐nm laser, the fluence was calibrated so that slight petechiae would be observed. The treatment consisted of a single‐pass application with no pulse overlap. Posttreatment care included the application of 0.3% prednisolone valerate acetate cream twice daily for 5 days. Sun protection measures were consistently recommended, although patient adherence varied.

### 2.3 Evaluation

Treatments were administered at 2‐month intervals, with patients returning for follow‐up at 2 weeks and 2 months post‐procedure. Digital images were captured under standardized conditions (light source, room, and camera) using a Canon E30 digital camera (Canon Inc., Tokyo, Japan) before and after each treatment session. Independent objective evaluations of pre‐ and posttreatment images were conducted by two clinicians blinded to the image sequence and treatment parameters using a 4‐point scale based on the degree of pigment clearance (excellent: 76%–100% improvement; good: 51%–75% improvement; fair: 26%–50% improvement; and poor: 1%–25% improvement). Patient‐reported improvement (satisfaction) was similarly evaluated at the 2‐month follow‐up using the same 4‐point scale (excellent, good, fair, and poor). Additionally, changes in melanin levels were assessed on the same day using VISIA skin analysis software (Canfield Scientific, Parsippany, NJ) in melanin mode, with results graded on a 4‐point scale (excellent: improvement ≥ 5; good: improvement 3–5; fair: improvement 1–3; and poor: improvement < 1). The VISIA skin analysis computer system uses a special numerical algorithm to determine the condition of the skin in an objective and reproducible way. The imaging device consists of a high‐resolution camera and polarized lighting, which are placed in a special capsule to ensure optimal and reproducible lighting conditions. The data are transmitted to a computer with VISIA software (version 6.3.4) installed. This system uses RBX technology (red/brown subcutaneous analysis) to identify the number of hyperpigmented lesions (brown areas) that exceed the reference value and to analyze the status (score) of these lesions. The software then analyzes and compares the target patient with a group of the same age and skin phototype in the database. In all evaluation methods, “effective” was defined as excellent or good. Adverse effects, including scarring, hypopigmentation, and hyperpigmentation, were assessed and documented. Pigmentary changes and scarring were graded on a four‐point scale (0 = none; 1 = mild; 2 = moderate; and 3 = severe hyperpigmentation, hypopigmentation, or scarring). To determine whether the deterioration of the ADM was due to PIH rather than ADM, we used objective evaluation by visual inspection and dermoscopy to determine whether there was an increase in pigmentation that differed from the original ADM color.

## 3 Results

### 3.1 Demographics

A total of 161 patients (all Asian females) participated in the study. Seventy‐eight and 83 patients were included in the 1064‐ and 730‐nm groups, respectively, with an average age of 34.3 years. The patient demographic characteristics are summarized in Table 1. Most patients had Fitzpatrick skin types III–IV (95.7%). Melasma was diagnosed in 64.6% of the study population. No significant demographic differences were observed between groups. The average treatment parameters are listed in Table 2.

**TABLE 1:** Patients' demographic characteristics.

Patient demographics	1064 nm	730 nm
Number	78	83
Age (years)	33.5	35.1
Fitzpatrick skin type		
I	0	0
II	0	0
III	29	28
IV	47	50
V	2	1
VI	0	0
Melasma	54 (69.2%)	50 (60.2%)

**TABLE 2:** Treatment parameters.

Treatment parameters	Spot size (mm)	Pulse duration (ps)	Fluence (J/cm^2^)
1064 nm	3	339	3.53 ± 0.25 [3.4–4.0]
730 nm	3	246	1.75 ± 0.05 [1.6–1.8]
	2		3.02 ± 0.12 [2.5–3.25]

### 3.2 Treatment Results

All the patients tolerated the treatment well. Table 3 shows the results based on the data collected at the final visit, 2 months after the end of the three sessions. After three sessions, the efficacy in terms of objective findings was 59.0% for the 1064‐nm group versus 88.0% for the 730‐nm group, patient‐reported satisfaction was 47.4% versus 93.9%, and VISIA analysis showed 60.3% versus 95.2% improvement, all favoring the 730‐nm group (Table 3). The incidence of hyperpigmentation was 15.4% in the 1064‐nm group and 14.5% in the 730‐nm group. When analyzing pigmentation in patients with melasma, all 12 patients who experienced hyperpigmentation in the 1064‐nm group (100%) had melasma, whereas the corresponding value for the 730‐nm group was only 58.3%. No other complications were observed, or they were very rare, with no significant differences between groups (Table 4).

**TABLE 3:** Result of treatment.

**Patient satisfaction**	**Excellent**	**Good**	**Fair**	**Poor**	**Effective (%)**
1064 nm	9	28	23	18	47.4
730 nm	23	55	3	2	94.0
**Objective evaluation**	**Excellent**	**Good**	**Fair**	**Poor**	**Effective (%)**
1064 nm	12	34	17	14	59.0
730 nm	33	40	8	2	88.0
**Software evaluation**	**Excellent**	**Good**	**Fair**	**Poor**	**Effective (%)**
1064 nm	10	37	19	12	60.3
730 nm	30	49	4	1	95.2

**TABLE 4:** Complications.

Complications	1064 nm		730 nm	
	All	Melasma	All	Melasma
Hyperpigmentation	12/78 (15.4%)	12/12 (100%)	12/83 (14.5%)	7/12 (58.3%)
Hypopigmentation	1/78 (1.3%)	1/1 (100%)	0/83 (0.0%)	0/0 (0.0%)
Texture change	1/78 (1.3%)	0/0 (0.0%)	0/83 (0.0%)	0/0 (0.0%)
Scar formation	0/78 (0.0%)	0/0 (0.0%)	0/83 (0.0%)	0/0 (0.0%)

### 3.3 Case 1

**FIGURE 1:**
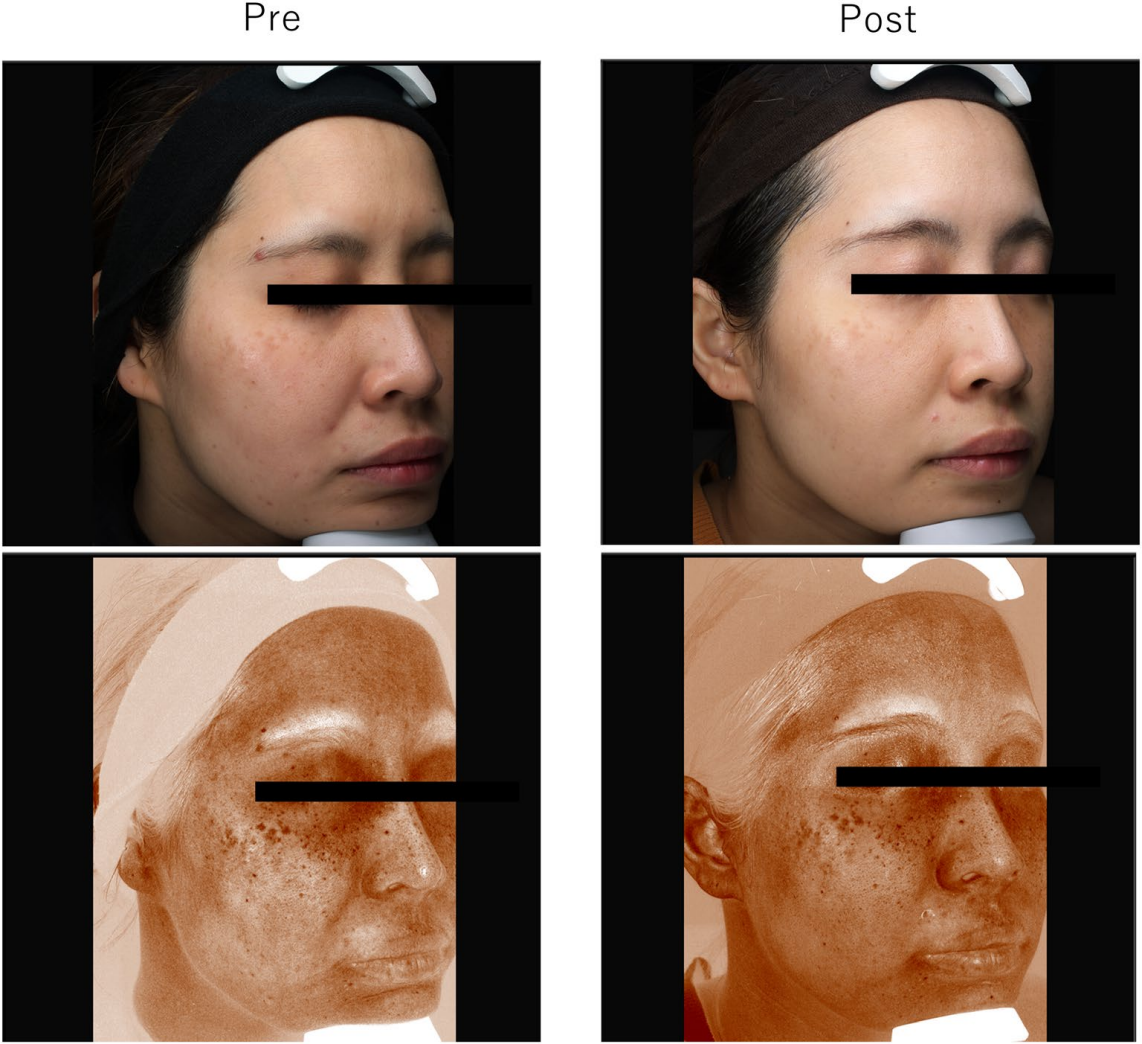
Case 1: A 28‐year‐old woman with acquired dermal melanocytosis on her cheeks without melasma was treated three times with a 1064‐nm picosecond laser.

A 28‐year‐old woman with ADM on the cheeks without melasma underwent three treatments with a 1064‐nm picosecond laser (3‐mm spot, 3.7 J/cm^2^ for the first session, 4.0 J/cm^2^ for subsequent sessions). Posttreatment objective evaluation, patient satisfaction, and image analysis (32 → 30) were all classified as poor (Figure 1).

### 3.4 Case 2

**FIGURE 2:**
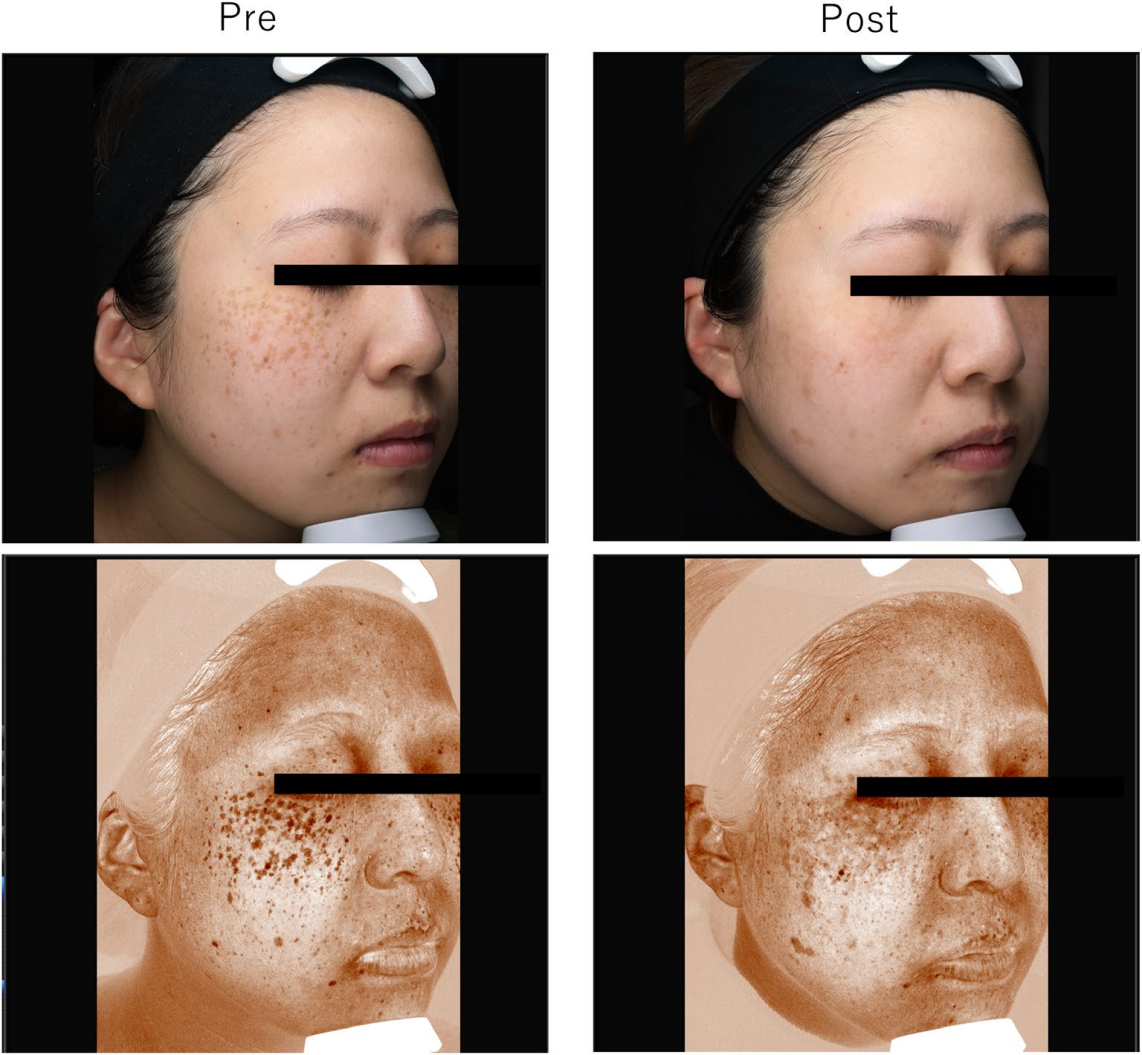
Case 2: A 30‐year‐old woman with acquired dermal melanocytosis on her cheeks without melasma was treated with a 730‐nm picosecond laser.

A 30‐year‐old woman with ADM on the cheeks without melasma underwent three treatments with the 730‐nm picosecond laser (3‐mm spot, 1.8 J/cm^2^ for all sessions). Posttreatment evaluations were good for objective evaluation and excellent for patient satisfaction and image analysis (28 → 23). In addition to the original ADM spot, a circular, light‐colored pigment spot was observed, which is thought to represent post‐inflammatory hyperpigmentation associated with the treatment (Figure 2).

### 3.5 Case 3

**FIGURE 3:**
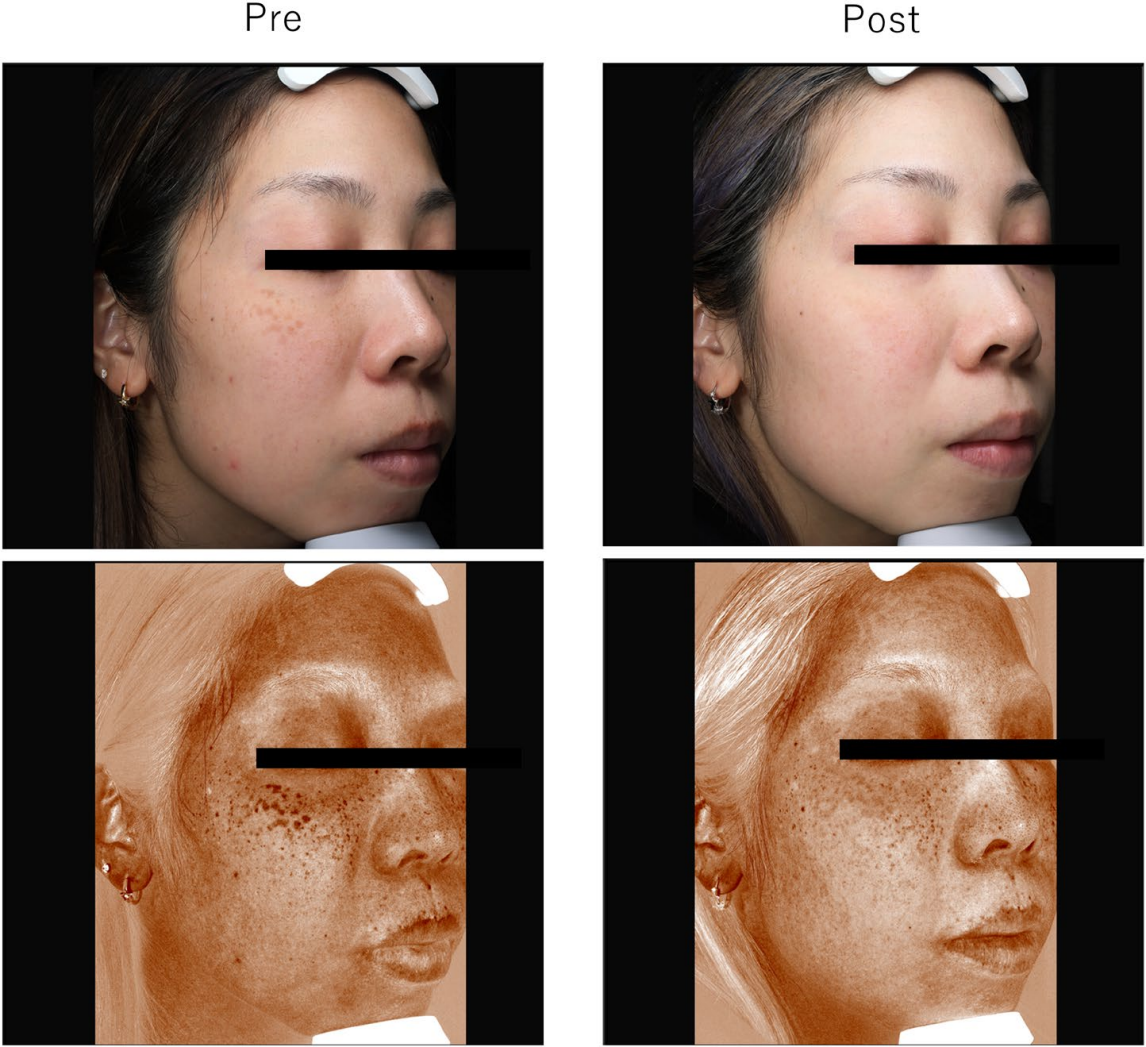
Case 3: A 38‐year‐old woman with acquired dermal melanocytosis on her cheeks and melasma was treated with a 730‐nm picosecond laser.

A 38‐year‐old woman with ADM on the cheeks with melasma underwent three treatments with the 730‐nm picosecond laser (3‐mm spot, 1.7 J/cm^2^ for the first session, 1.8 J/cm^2^ for subsequent sessions). Posttreatment evaluations revealed excellent outcomes for objective evaluation, patient satisfaction, and image analysis (43 → 37) (Figure 3).

### 3.6 Case 4

**FIGURE 4:**
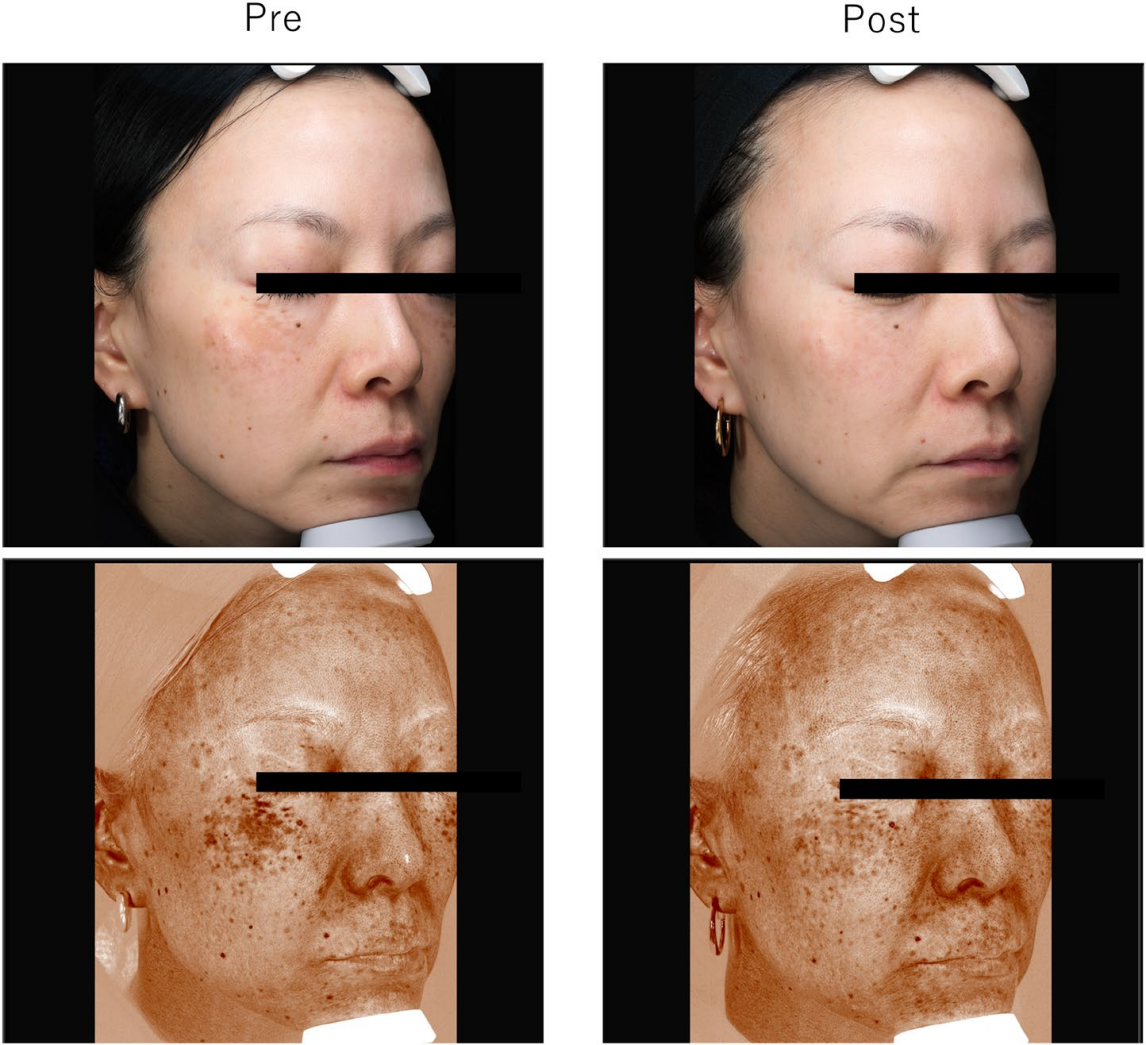
Case 4: A 44‐year‐old woman with acquired dermal melanocytosis on her cheeks and melasma was treated with a 730‐nm picosecond laser.

A 44‐year‐old woman with ADM on the cheeks with melasma underwent three treatments with the 730‐nm picosecond laser (3‐mm spot, 1.8 J/cm^2^ for all sessions). Posttreatment evaluations revealed excellent outcomes for objective evaluation, patient satisfaction, and image analysis (48 → 33) (Figure 4).

## 4 Discussion

Treating pigmentary disorders in patients with non‐white skin types, including Asians, remains challenging due to the increased risk of laser treatment‐related adverse effects, and consensus protocols are yet to be established. The potential side effects and relative paucity of evidence regarding ADM laser treatment in this population often result in prolonged treatment durations and frequent incomplete clearance. For over 20 years, Q‐switched lasers have played a major role in the treatment of ADM. However, while high‐fluence treatments for early removal produce very high post‐inflammatory hyperpigmentation, low‐fluence treatments require a long treatment period with multiple treatment sessions, which places a burden on patients both financially and mentally [14, 15]. Considering these challenges, further research is needed to identify the optimal treatment modalities and parameters for pigmentary disorders, including ADM, in patients with non‐white skin. However, the relevant evidence base is limited. To the best of our knowledge, this is the first retrospective study to demonstrate the efficacy of a 730‐nm picosecond laser in treating ADM, particularly in patients with non‐white skin. The data showed that the 730‐nm wavelength picosecond laser offers significant cosmetic benefits for treating ADM in Asian patients, with high tolerability and improved results with fewer treatment sessions. Various studies have shown that different picosecond lasers (532, 755, 785, and 1064 nm) are safe and effective in treating specific benign pigmented lesions, such as solar lentigines, freckles, café‐au‐lait spots, flat and raised epidermal lesions, Becker's nevus, flat nevi, nevus of Ota, Hori's nevus, and verrucous epidermal nevi [16, 17, 18, 19, 20, 21]. Theoretically, for the selective destruction of a target, the laser energy must be at a wavelength that is highly absorbed by the target relative to the surrounding tissue, and the pulse duration must be shorter than the time required for heat to dissipate through conduction [22]. Furthermore, based on the theory of stress relaxation time, the shorter the pulse duration, the more likely the primary response is to involve photomechanical disruption rather than photothermal effects [23]. The effectiveness of continuous use of 755‐nm alexandrite and/or 1064‐nm neodymium: yttrium aluminum garnet lasers has been reported for ADM treatment, but the appropriate wavelength has not been fully discussed [20]. In terms of wavelength, shorter wavelengths in the visible light spectrum are more strongly absorbed by melanin, suggesting that a 730‐nm wavelength could be more effective in removing unwanted pigmentation, such as lentigines and freckles, compared to 785‐ or 755‐nm wavelengths [24]. The laser used in this study had a pulse width of 246 ps; theoretically, the combination of a 730‐nm wavelength with the very short pulse width of 246 ps allowed for more selective destruction of melanin and melanosomes with a higher safety margin. ADM is histopathologically characterized by the presence of melanocytes in the upper to mid‐dermis with relatively immature melanosomes, which may explain the efficacy of the 730‐nm wavelength treatment [25].

One of the most likely complications of laser treatment is PIH. The estimated PIH rate for treating benign lentigines with picosecond lasers ranges from 5% to 10%. In particular, the incidence rates in the few reports on 730‐nm wavelength picosecond laser were lower than in our study, at 3.4% (192 ± 145 days intervals) and 8.33% (single session) [26, 27]. Considering the inter‐study variability, this difference may be due to the induction of inflammation by the short treatment interval of 8 weeks, or the relatively high underlying prevalence of melasma in the study population, in addition to natural variations.

In cases where ADM and melasma coexist, laser treatment prevents the progression of ADM, but histologically, it has been shown that the worsening of pigmentation that occurs is due to the induction of melasma [28]. Interestingly, all patients in the 1064‐nm group who showed hyperpigmentation were those who also had melasma and ADM, whereas in the 730‐nm group, there were cases where hyperpigmentation was not observed even when melasma was present. Previous reports have shown that treatment of ADM with a 1064‐nm Q‐switched laser can worsen melasma, and it is possible that the 12 cases confirmed in this study showed increased pigmentation as a worsening of melasma rather than a recurrence of ADM [27]. On the other hand, reports of melasma improvement with low‐fluence 730‐nm laser irradiation support the fact that there were multiple cases in our study where we did not observe hyperpigmentation due to melasma aggravation [29]. It has also been reported that the use of a 730‐nm wavelength does not worsen melasma [30]; however, in this study, the treatment was carried out at a higher fluence than that used for melasma treatment, so there is a possibility that it caused mild inflammation of the skin unrelated to melasma, leading to pigmentation.

In some cases, PIH was observed despite the application of a topical steroid after the procedure. This is thought to be due to the fact that the wavelength and target chromophore of the laser used in this study both reach the dermis, while epidermal pigmentation usually resolves or improves significantly within 6–12 months, but dermal pigmentation improves more slowly and can be permanent [31].

The limitations of this study include the exclusive focus on Asian women, the restricted number of treatment sessions, insufficient evaluation of long‐term effects, and the fact that there was only one type of protocol with a 2‐month interval. Furthermore, as the diagnosis of ADM was made based only on clinical findings and no biopsies were performed, there is a possibility that some patients included in the study had other pigmented lesions that were not ADM. In cases where ADM and melasma are combined, it is possible that melasma is improving at the same time as the ADM pigmented spots, and the data from this study may show the effectiveness of the treatment for both lesions. Additionally, while PIH typically occurs within 4 weeks post‐laser treatment, some individuals may develop PIH later. Furthermore, most patients experience recurrence within 3–6 months post‐laser treatment, necessitating longer follow‐up periods [32].

The use of the 730‐nm picosecond laser, which exhibits high melanin selectivity, shorter pulse length, and relatively deep penetration, suggests that it may effectively treat ADM without increasing complications compared to the conventional 1064‐nm wavelength.

## Ethics Statement

This study was conducted according to the principles of the Declaration of Helsinki. The study protocol was reviewed and approved by the Institutional Review Board (IRB) of Etoile Regina Clinic. Written informed consent was obtained from the parents prior to study participation, including consent to participate and to publish the findings.

## Conflicts of Interest

The authors declare no conflicts of interest.

## Data Availability

Data supporting the findings of this study are available from the corresponding author, K.T., upon reasonable request.
